# High-density linkage mapping and evolution of paralogs and orthologs in *Salix *and *Populus*

**DOI:** 10.1186/1471-2164-11-129

**Published:** 2010-02-23

**Authors:** Sofia Berlin, Ulf Lagercrantz, Sara von Arnold, Torbjörn Öst, Ann Christin Rönnberg-Wästljung

**Affiliations:** 1 Department of Plant Biology and Forest Genetics, Uppsala BioCenter, Swedish University of Agricultural Sciences, PO Box 7090, SE-750 07 Uppsala, Sweden; 2 Department of Evolutionary Functional Genomics, Evolutionary Biology Centre, Uppsala University, Norbyvägen 18 D, SE-752 36 Uppsala, Sweden; 3 Department of Medical Sciences, Uppsala University, Uppsala University Hospital, SE-751 85 Uppsala, Sweden

## Abstract

**Background:**

*Salix *(willow) and *Populus *(poplar) are members of the Salicaceae family and they share many ecological as well as genetic and genomic characteristics. The interest of using willow for biomass production is growing, which has resulted in increased pressure on breeding of high yielding and resistant clones adapted to different environments. The main purpose of this work was to develop dense genetic linkage maps for mapping of traits related to yield and resistance in willow. We used the *Populus trichocarpa *genome to extract evenly spaced markers and mapped the orthologous loci in the willow genome. The marker positions in the two genomes were used to study genome evolution since the divergence of the two lineages some 45 mya.

**Results:**

We constructed two linkage maps covering the 19 linkage groups in willow. The most detailed consensus map, S_1_, contains 495 markers with a total genetic distance of 2477 cM and an average distance of 5.0 cM between the markers. The S_3 _consensus map contains 221 markers and has a total genetic distance of 1793 cM and an average distance of 8.1 cM between the markers. We found high degree of synteny and gene order conservation between willow and poplar. There is however evidence for two major interchromosomal rearrangements involving poplar LG I and XVI and willow LG Ib, suggesting a fission or a fusion in one of the lineages, as well as five intrachromosomal inversions. The number of silent substitutions were three times lower (median: 0.12) between orthologs than between paralogs (median: 0.37 - 0.41).

**Conclusions:**

The relatively slow rates of genomic change between willow and poplar mean that the genomic resources in poplar will be most useful in genomic research in willow, such as identifying genes underlying QTLs of important traits. Our data suggest that the whole-genome duplication occurred long before the divergence of the two genera, events which have until now been regarded as contemporary. Estimated silent substitution rates were 1.28 × 10^-9 ^and 1.68 × 10^-9 ^per site and year, which are close to rates found in other perennials but much lower than rates in annuals.

## Background

Genomic analyses of related organisms are central for addressing the evolution of genome organisation as well as for supporting the identification of the genetic background of economically and biologically important traits. With genetic linkage maps and genome sequence information available across taxa, it is possible to study evolutionary processes ranging from major structural changes like whole-genome duplications and chromosome rearrangements to fine scale differences such as single base substitutions. The rate of chromosomal rearrangements differs widely both among and within eukaryotic lineages [1-4]. Although the exact mechanisms creating rearrangements are unknown, repetitive elements seem to be an important trigger as breakpoint regions of rearrangements are enriched in various classes of repeats [1]. Changes in chromosome numbers may occur through fission and fusion of chromosomes, or more radically through polyploidization (genome duplication). The highly variable rate observed for gross chromosomal rearrangements in different lineages is also evident at the nucleotide substitution level [5]. Base substitutions at neutral sites occur at a rate equivalent to the mutation rate. This rate is affected by the number of mitotic cell divisions in animal germ lines or in plant cell lines ultimately forming gametes, during which replication errors can cause mutations. With information on divergence times obtained from the fossil record and estimates of the number of neutral substitutions in orthologous genes (K) one can through the relationship, r = K/(2T) [5] estimate neutral substitution rates (r). Although there is uncertainty in the dating of divergence times using the fossil record, this method gives rough estimates of the rate of silent substitutions in the species under study. In addition, the formula can be applied for studying relative timing of speciation and genome duplication events by comparing the number of substitutions in paralogous and orthologus genes, assuming constant substitution rates through evolutionary time. It is generally thought that orthologs share similar functions whereas paralogs often have different functions, and therefore paralogs are expected to diverge more per unit time than orthologs. This has however recently been questioned as orthologs might obtain new functions at a similar rate as paralogs [6]. Furthermore, functional change is not expected to affect silent substitution rates.

*Salix *and *Populus *species are trees, shrubs or sub-shrubs and members of the Salicaceae family. They share many characteristics such as dioecy, rapid growth and seed development, and ease with which they can be vegetatively propagated. Species across both genera typically have a haploid chromosome number of 19 and small and convenient genomes sizes (~500 Mbp) compared to the large genomes of most conifers (15 - 35 Gbp). Based on the fossil record, the divergence of the two genera has been dated to approximately 45 mya [7,8]. *Populus *is recognized as a model genus for genetic and genomic studies in angiosperm trees, with many resources available such as the genome sequence of *Populus trichocarpa *http://genome.jgi-psf.org/Poptr1_1/Poptr1_1.home.html, [9], linkage maps [10-15] and microarrays [16,17].

The genus *Salix *comprises more than 300 species and is widespread in both the Northern and the Southern hemisphere, excluding Australasia and New Guinea. Many *Salix *species display rapid growth and high biomass yields, even when nitrogen fertilizers are used sparsely [18]. These characteristics together with ease of establishment and a broad range of adaptability explain the wide-spread use of *Salix *spp. for short rotation biomass production. The large phenotypic variation within and among *Salix *species facilitates the identification of quantitative trait loci (QTLs) in experimental populations. Also, natural populations of *Salix *typically display high genetic diversity and are likely to contain many low-frequency alleles that underlie outstanding phenotypes useful to breeders [19]. *Salix viminalis*, *S. dascyclados *and *S. schwerinii *and their hybrids are some of the most commonly used *Salix *species in the breeding programs in Europe. These species have also been the focus of most past investigations of *Salix *genetics, which include the generation of linkage maps [20-24] and QTL analyses i.e. frost resistance and phenology [25,26], growth, water-use efficiency and drought tolerance [27,28]. As the use of willow as a source of biomass is expected to increase in the near future (S. Larsson Lantmännen Agroenergi AB, personal communication), one can foresee a rising demand for high yielding willow clones that are adapted to different environments and resistant to a wide repertoire of pathogens. This is going to put more pressure on breeding activities and more efficient ways to select useful clones will be needed. One method that can strongly support these activities is "Marker Assisted Selection" (MAS), i.e. selection on easily detectable genetic markers in genes linked to QTLs underlying phenotypic traits. MAS should be particularly useful for traits that are difficult to measure, exhibit low heritability, and/or are expressed late in development, such as productivity, disease resistance, drought and heat tolerance. To develop efficient MAS in willow, QTLs and ultimately genes responsible for selected phenotypic traits must first be identified. Quantitative traits are expected to have complicated genetic backgrounds including many genes, and complex interactions between them. Mapping such traits requires dense linkage maps, large mapping populations and thorough phenotyping of plants grown both in greenhouse and in field experiments over multiple seasons.

In this study we present two willow genetic linkage maps based on two mapping populations with the major aim of investigating the degree of genomic conservation between willow and poplar (*Populus*). The first more detailed map (S_1_) is based on a *S. viminalis *× (*S. viminalis *× *S. schwerinii*) cross using SNP, microsatellite and AFLP markers. The second map (S_3_) is based on a *S. viminalis *× *S. viminalis *cross and SNP and microsatellite markers. Both maps are considerably more detailed than previously available willow linkage maps, and in contrast to those they are to a large extent based on SNP markers with a genome-wide distribution for which the corresponding positions in the poplar genome are known. It was therefore possible to investigate the degree of synteny and gene-order conservation across a significant part of the two Salicaceae genomes by comparing the willow linkage maps with the physical map constructed from the poplar genome sequence. This is a useful approach for studying genome evolution between less well studied species and model plant species with sequenced genomes. In addition, we estimated the number of substitutions in introns (K_i_) between orthologous and paralogous genes in willow and poplar, and used the distributions of these estimates to infer the relative age of the divergence between the lineages leading to *Salix *and *Populus *and age of the whole genome duplication shared by these lineages.

## Results

### SNP markers and genotyping

In total, 426 willow gene fragments containing SNPs were evaluated for genotyping at the SNP Technology Platform (Uppsala University). Of these 426 SNPs, 350 were identified using primers developed for the purpose of this study, representing 309 different genes, hence, a number of genes contained multiple SNPs. 12 SNPs were previously identified in phenology candidate genes (N. Gyllenstrand, personal communication) (Additional file 1) and 64 SNPs (in 58 unique genes) were identified using primers from Hanley *et al*. (2006). See Additional file 2 for marker information and primer sequences. Homology searches against the poplar genome with the 309 willow sequences developed in this study indicated a most significant hit at the position of the expected target in all but 24 instances (Table 1). In 20 of these cases, the willow sequence was more similar to the paralogous gene copy (paralog 2) than to the orthologous gene expected to be amplified (paralog 1). Furthermore, the positions in the willow genome match the positions of paralog 2 in the poplar genome in all but one case (XI_14om), suggesting that the paralogs were erroneously amplified and sequenced in willow. XI_14om is unique in the sense that based on sequence homology, the willow sequence is most similar to paralog 2 in poplar, however the position in the willow genome does not agree with the position of paralog 2 in poplar. In the other four cases, the willow sequences showed best sequence homology to genomic regions other than the expected (Table 1).

**Table 1:** Markers representing cases when the willow sequence shows best sequence homology to the poplar paralog (paralog 2) or to some other genomic region in poplar other than the expected (paralog 1).

Marker	Poplar paralog 1*	Poplar paralog 2**	Willow ***	Willow LG
XI_19_sa	XI:13708412- 13713847	I:34332298- 34337706	I:34336820- 34337014	Ib
VI_21_sa	VI:1059584- 1063805	XVI:175981- 180636	XVI:179650- 180165	Ib
XI_14om_sa	XI:13390381- 13396292	XIII:8162057- 8167721	XIII:8162846- 8163488	Ib
XVII_8om_sa	XVII:2862545- 2865794	I:21917969- 21921284	I:21918035- 21918509	Ib
V-3	V	NA	II: II:7149915- 7150175	II
XIV_2om_sa_pI+pIII	XIV:1405173- 1410344	II:11032656- 11037852	II:11034560- 11035232	II
I-3_sa	I:2799329- 2802489	III:16679089- 16681782	III:16679770- 16679455	III
II_27_sa	II:659865- 663083	V:17347198- 17350454	V:17347673- 17348033	V
X_23_sa_pI	X:2909535- 2910774	VIII:13507022- 13508215	VIII:13507309- 13508014	VIII
X_5_sa_pI+pIII	X:4387251- 4392821	scaff.132:44316- 50181	scaff.132:46692- 47354	VIII
I_55_sa	I:19888722- 19894389	IX:6182711- 6188752	IX:6188219- 6188751	IX
I_56_sa	I:20786788- 20790053	IX:5366669- 5370261	IX:5368352- 5368805	IX
R_79_sa	VIII:2675046- 2678768	X:18365204- 18369371	X:18368575- 18368938	X
I-64_sa	I:28954163- 28960895	XI:5889680- 5902742	XI:5899792- 5899792	XI
XV_14_sa	XV:4861609- 4865109	XII:9029417- 9031788	XII:9030854- 9031468	XII
XV_3om_sa	XV:2214022- 2229691	XII:5650497- 5668349	XII:5659107- 5659662	XII
XIX_16_sa	XIX:10024624- 10029438	scaff.142:594094- 598587	scaff.142:595203- 595639	XIII
II-22_sa	II:20278259- 20280545	XIV:7371540- 7374037	XIV:7372638- 7372921	XIV
II-15_sa	II:13716352- 13719221	XIV:3952304- 3955719	XIV:3952744- 3952880	XIV
R-76_sa	XIII:13007808- 13016472	XIX:10828344- 10837472	XIX:10828231- 10828870	XIX
Cases when the willow fragment shows best sequence homology to a different region than paralog 1 and 2 in poplar				
*I-44_sa*	*I:8108096- 8110817*	*III:11697654- 11701038*	*I:28482702- 28483096*	*Ib*
*VI_20_sa*	*VI:175694- 177367*	*I:24601135- 24605961*	*scaff.137:193270- 193790*	*Ib*
*XVI-4_sa*	*XVI:3509806- 3516550*	*VI:3514697- 3520404*	*XVI:3668954- 3669420*	*Ib*
*VI-22_sa*	*VI:9566935- 9572156*	*scaff.215:114354- 120054*	*IV:16209337- 16209466*	*B*

* Genomic region in poplar where primers were designed

** The second best BLAST hit with paralog 1

*** Best BLAST hit with the willow sequence

NA = no alignment available since the markers were developed by Hanley et al. (2006)

396 SNPs were selected and genotyped in the S_1 _and S_3 _mapping populations. In a first panel of 384 SNPs conversion rate was 92% (354/384) and the overall sample call rate for the approved SNPs was 97.5%. The reproducibility was 100% according to duplicate analysis of 2.3% of the genotypes. In the second 12 SNP panel, the SNP conversion rate was 83% (10/12) and the overall sample call rate for the approved SNPs was 99.0%. The reproducibility was 100% according to duplicate analysis of 39.5% of the genotypes.

### Linkage mapping

**Figure 1:**
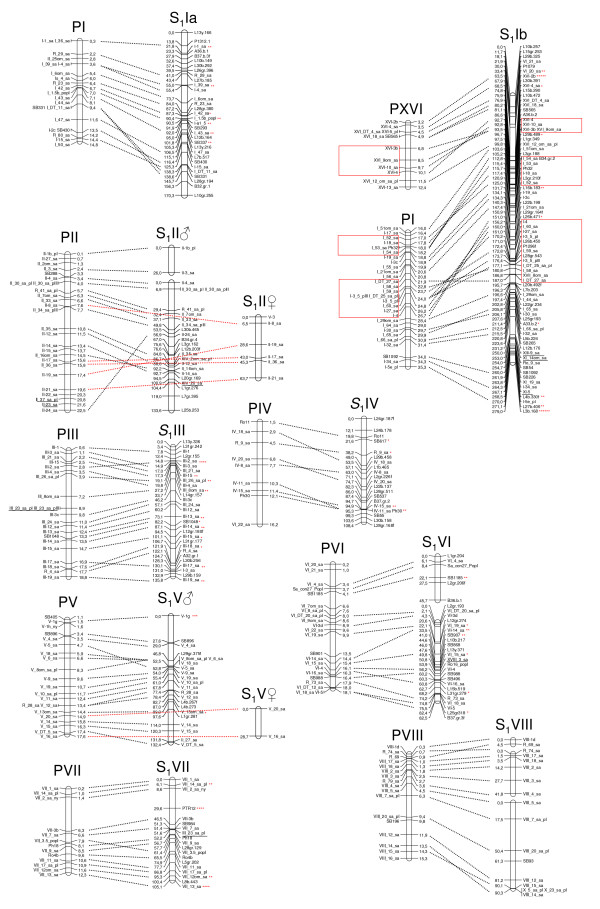
**The first eight linkage groups (LGI - LGVIII) of the consensus S_1 _linkage map aligned to the poplar physical map**. The willow consensus S_1 _linkage map aligned to the poplar genomic sequence (prefixed with P) based on SNP, microsatellite and AFLP markers with approximate positions of markers given in base pairs (bp) and centimorgans (cM) respectively. Loci that may indicate syntenic disparities are underlined. Markers showing segregation distortion are indicated by asterisks on the willow map (one asterisk: P < 0.1, two asterisks: P < 0.05, three asterisks: P < 0.01: four asterisks: P < 0.005, five asterisks: P < 0.001, six asterisks: P < 0.0005, seven asterisks: P < 0.0001). Three inversions are indicated by boxes.

**Figure 2:**
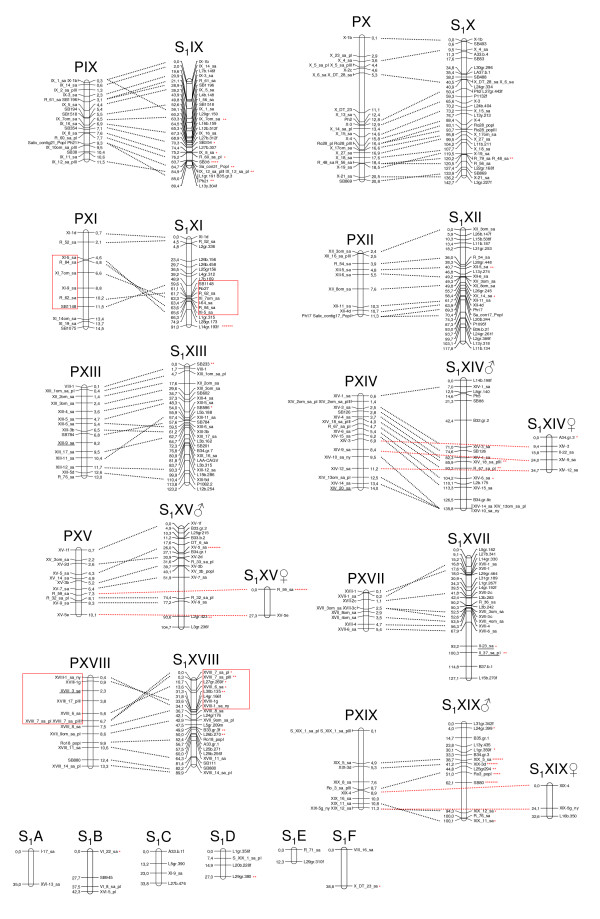
**The last eleven linkage groups (LGIX - LGXIX) of the consensus S_1 _linkage map aligned to the poplar physical map**. The willow consensus S_1 _linkage map aligned to the poplar genomic sequence (prefixed with P) based on SNP, microsatellite and AFLP markers with approximate positions of markers given in base pairs (bp) and centimorgans (cM) respectively. Loci that may indicate syntenic disparities are underlined. Willow linkage groups S_1_A to S_1_F that could not be aligned to the poplar genome are also shown. Markers showing segregation distortion are indicated by asterisks on the willow map (one asterisk: P < 0.1, two asterisks: P < 0.05, three asterisks: P < 0.01: four asterisks: P < 0.005, five asterisks: P < 0.001, six asterisks: P < 0.0005, seven asterisks: P < 0.0001). Two inversions are indicated by boxes.

After excluding SNPs that were monomorphic or contained null alleles, segregation data for 307 SNPs in 463 individuals were used to construct linkage map S_1 _(see Additional file 2). 75 markers were heterozygous only in the female parent, 158 only in the male parent and 74 were heterozygous in both. In addition, 45 microsatellite markers were included, of which 25 segregated in both parents, six were maternally informative and 14 were paternally informative. Finally, 287 AFLP markers were included, with genotypic data on 89 individuals. Consensus linkage maps were constructed using all 639 markers together. In the *LOD Groupings *step in Joinmap, markers were grouped at a LOD threshold of 5.0 and 25 linkage groups were identified of which three were duplets and three contained only four markers. The other 19 groups represent the 19 linkage groups in poplar (Figure 1 and Figure 2). 48 AFLP markers were unlinked and excluded from further analysis. For the markers within each group, map calculations were performed in Joinmap based on recombination frequencies, which is the step when linked markers are placed on linkage groups and ordered relative to each other. For five groups (II, V, XIV, XV and XIX) no consensus LGs could be calculated due to lack of markers segregating in both parents. These groups were split into the parental groups and the markers tested separately within each parental group. A number of markers remained unlinked after the map calculations due to insufficient linkage to the other markers. These markers were tested separately within groups to examine if any of them were linked to each other. A number of small groups were defined in this way and included in the map. Ten of the groups (eight duplets and two with four markers) contained only AFLPs and since they cannot be connected to the poplar genome they were excluded from further discussion. After inspection of LG VI, marker ph27 was removed because of inconsistency in the placement of this marker compared to S_3 _and poplar.

In general, the S_1 _map without the AFLP markers was in very good agreement with the map including the AFLP markers. However, in LG VIII the gene order differed slightly when constructing the map with or without the AFLP markers. Since the gene order in this linkage group without the AFLP markers was in better agreement with the gene order in the poplar genome as well as with the S_3 _map, we present LG VIII without AFLP markers in Figure 1 (all other LGs in Figure 1 and Figure 2 include AFLP markers). Furthermore, II-24_sa on LG II was not mapped when no AFLP markers were included. 88 markers in map S_1 _showed distorted segregation ratios (P < 0.00001). XI_21_sa and R_66_sa were removed because they were distorted and had a high number of missing genotypes. The consensus S_1 _linkage map with 495 linked markers aligned to the poplar physical map is shown in Figure 1 and Figure 2. The map spanned 2477 cM with an average interval between the markers of 5.0 cM. The linkage groups A to F and the female linkage groups present on LGs II, V, XIV, XV and XIX, where no consensus maps were constructed are not included in the total map length.

214 SNPs genotyped in 282 individuals were used to construct the linkage map S_3. _94 SNPs segregated in the female parent only, 79 in the male parent only, and 41 in both parents. 41 microsatellite markers were also included, 21 of which segregated in both parents, 13 were maternally informative and seven were paternally informative (Additional file 2). Consensus linkage maps were constructed using all 255 markers. For map S_3_, marker groupings at a LOD threshold of 4.0 identified 27 linkage groups and seven markers remained unlinked. Similarly to S_1_, some markers that did not map to the other markers within linkage groups due to insufficient linkage were tested separately. Groups Ia, Ib, V, VII, VIII, IX, XII, XIV, XV, XVII and XVIII are represented by more than one linkage group in S_3_, and they were placed on the map based on positional information in S_1 _(Additional file 3). The most plausible reasons why we were unable to detect linkage between the small groups and other linkage groups is likely an effect of the small mapping population and limited number of markers, and as a consequence markers flanking the gaps can be too far apart, contain too little information, or are separated by a region of high recombination rate. Distorted segregation ratios (p < 0.00001) were found for 38 markers. R_24_sa and XVI_5_pIII form a separate duplet that cannot with certainty be connected to any LG. The same applies to the group containing SB945 and VI_8_sa_pIII.

There are no major rearrangements distinguishing the two willow maps, although there are a few apparent discrepancies in marker order on some LGs. Most differences involve closely spaced markers located on small groups or at the distal ends of groups, where the exact positioning of markers is uncertain. In several cases there was a swop in the positioning of two markers with tiny genetic distances, most likely an effect of the small size of the S_3 _mapping population. The S_3 _consensus map, excluding the small groups S_3_A and S_3_B and the groups S_3_Ia-2 and S_3_V-2 that could not be placed in groups Ia and V respectively, consists of 221 markers aligned to the S_1 _map and is shown in Additional file 3. S_3 _spanned 1793 cM with an average interval between the markers of 8.1 cM.

We could link nine previously unmapped scaffolds in poplar to linkage groups in the willow genome (scaffold 64 and 134 to LG Ib + XVI, scaffold 88 to LG V, scaffold 132 to LG VIII, scaffold 899 to LG XI, scaffold 142 to XIII, scaffold 40 to LG XIV and scaffold 82 and 122 to LG XV). We were also able to merge some groups that were unlinked in Hanley *et al*. (2006) (IIIa and IIIb, VIb and VIc, VIIb and VIIc, IXa and IXb, XIIIa and XIIIb, XVIIa and XVIIb, XIXa and XIXb).

### Comparative mapping

We found two major interchromosomal rearrangements distinguishing the karyotype of willow from poplar, involving poplar LG I and XVI and willow LG Ia and Ib. Markers located on poplar LG I are in willow placed on two different LGs; willow LG Ia aligns to the first part of poplar LG I, from 0.3 Mb to 14.8 Mb (Figure 1) and willow LG Ib aligns to markers on the second part of poplar LG I, from 16.0 Mb to 35.3 Mb. In addition, markers located on poplar XVI are in the willow map linked to LG Ib. These data support either a fusion resulting in poplar LG I or a fission resulting in willow LG Ia and Ib sometime since the divergence of the two lineages. Similarly, either a fission in the poplar lineage forming poplar LG I and XVI or a fusion in the willow lineage forming willow LG Ib must have happened since the divergence of the two species (Figure 1).

Seven markers in the willow map should based on sequence homology be true orthologs, but the position of the markers in the willow linkage map differs from the corresponding position in the poplar genome. These cases could possibly represent cases of translocation of limited chromosomal segments (Table 2).

**Table 2:** Markers representing cases where the willow sequence shows best sequence homology to the expected location in the poplar genome (paralog 1), but with a different position in the willow map than the expected, possibly representing translocations of small chromosomal segments between the genomes.

Marker	Poplar paralog 1*	Poplar paralog 2**	Willow ***	Willow LG
XI-5	XI	NA	XI:14789231- 14789785	Ib
XIII-9_sa	XIII:8154297- 8159215	No hit	XIII:8156663- 8157295	Ib
XIV_20_sa	XIV:13995907- 14001590	No hit	XIV:13996955- 13997593	II
XVIII_3_sa	XVIII:2310344- 2313479	No hit	XVIII:2312570- 2312814	VI
III_23_sa_pI+pIII	III:8892578- 8897817	No hit	III:8892660- 8893230	VII
II_37_sa_pI	II:20797060- 20804409	IV:7209830- 7217966	II:20803016- 20803505	XVII
II-23_sa	II:21561743- 21565690	No hit	II:21562039- 21562808	XVII

* Genomic region in poplar where primers were designed

** The second best BLAST hit with paralog 1

*** Best BLAST hit with the willow sequence

NA = no alignment available since the markers were developed by Hanley et al. (2006)

Generally, gene order within syntenic groups is very well conserved in the willow - poplar comparison. However, the data indicate a few differences between the two genomes, five of which are inversions involving several markers each. Three of these are located on willow LG Ib (Figure 1) and correspond in size to ~1.6 - 5.4 Mb in the poplar genome sequence. In poplar, these regions correspond to parts of LG XVI and I. The other two supported inversions are located on LG XI and XVIII and involve roughly half of the total lengths of the linkage groups (6.9 Mb on LG XI and 6.3 Mb on LG XVIII) (Figure 2). For LG XI, the inversion is not evident in the S_3 _map, which shows the same marker order as poplar, while the inversion on LG XVIII cannot be detected in the S_3 _map because the markers involved are located on a separate linkage group so the gene order cannot be determined. Apart from these supported differences there are a number of suggested gene order differences between the willow and the poplar genomes, it is however uncertain whether or not there are true differences or errors in either the poplar genome assembly or the willow linkage maps. The assembly of the poplar genome is not complete and most likely the genome sequence as it is presented today contains numerous gaps.

### Sequence analyses

**Figure 3:**
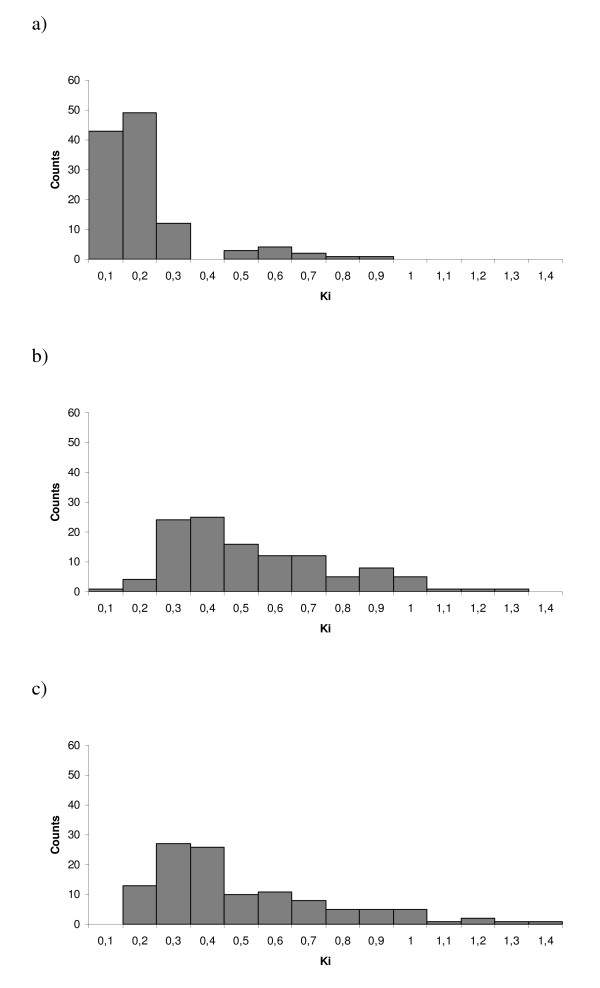
**The distributions of pairwise K_i _distances. K_i _values are grouped into bins of 0.1**. a) between willow and poplar orthologs. b) between willow and poplar paralogs. c) between poplar paralogs.

For 115 gene fragments where three sequences were present (willow, poplar paralog 1 and poplar paralog 2) alignments were constructed and used to estimate pairwise K_i _between orthologous loci in willow and poplar, paralogous loci in willow and poplar and paralogous loci in poplar (Figure 3). The willow sequence, the expected part of poplar paralog1 to be amplified and the total genomic sequence of poplar paralog 2 and default parameters were used to construct the alignments. A total of 48 kb genomic DNA sequence were used for estimations of K_i_. As can be seen in the distributions of orthologous K_i _(Figure 3), there are sequence pairs with high K_i _values (> 1). This is likely a result of erroneous alignments when gaps were present in one of the sequences, or if parts of the overlapping sequences were missing, resulting in alignments of non-homologous nucleotides. It is however clear that the majority of orthologous sequence pairs have K_i _between 0.1 and 0.2. Including all alignments, mean K_i _between orthologs in willow and poplar was 0.17 ± 0.01 and the median K_i _was 0.12. The distributions of K_i _values for paralogous loci show a peak in the number of sequence pairs around 0.3 - 0.4. Mean K_i _between paralogs in willow and poplar was 0.48 ± 0.02 and between the paralogs in poplar, 0.45 ± 0.02. The corresponding medians were 0.42 and 0.37. K_i _between orthologs were significantly lower than K_i _between paralogs in willow and poplar (Mann Whitney U test, W = 7900, p < 0.00001) and between paralogs in poplar (Mann Whitney U test, W = 8095, p < 0.00001). K_i _between the two estimates of paralogs were not significantly different (Mann Whitney U test, W = 14022, p = 0.1).

## Discussion

Based on our large sample of sequence based markers and large pedigrees we have studied genome evolution in species from two tree genera, *Salix *and *Populus *by constructing linkage maps in *Salix *and aligning them to the *Populus trichocarpa *genome sequence. The large number of SNP markers with known location and even distribution in the poplar genome allowed the first detailed comparison between the two genomes. We have used the physical information from the poplar genome to approximate the proportion of the willow genome covered by our maps. The size of the poplar genome has been estimated to 485 ± 10 Mb of which approximately 410 Mb has been assembled [9] and approximately 310 Mb have been assigned to linkage groups according to the poplar genome website. Our S_1 _linkage map covers about 280 Mb of the poplar genome or 90% of the DNA sequence assigned to linkage groups. Most likely this fraction (the assembled and assigned genomic sequence) of the poplar genome is mostly euchromatic DNA including the majority of transcribed genes. As much as 40% of the whole poplar genome consists of repetitive elements [29] and we assume that the majority of the unassembled genome sequence is heterochromatic DNA. This means that our maps cover most of the genic regions of the genome with less coverage in the heterochromatic regions.

The willow markers form 19 major linkage groups, as expected based on the haploid chromosome number of 19. Aligning the willow linkage map to the poplar genome, we find strong support for two interchromosomal rearrangements since the divergence of the two lineages. One rearrangement involve poplar LG I and willow LG Ia and Ib, and is either a result of a fusion in the poplar lineage or a fission in the willow lineage since the divergence from a common ancestor. The second rearrangement involve poplar LG I and XVI and willow LG Ib and is either a result of a fission in the poplar linage or a fusion in the willow lineage sometime since the divergence from a common ancestor. In support of our observations, Hanley *et al*. (2006) identified two groups in their willow map aligning to poplar LG I, but in that study, the willow LG aligning to poplar LG XVI was not linked to willow LG Ib as was the case in the present study. The correspondence between LG Ia and Ib in Hanley *et al*. (2006) and Ia and Ib in the present study is supported by three common markers on Ia and seven common markers on Ib. The most plausible reason why we found support for linkage of willow Ib and poplar XVI is the significantly higher number of SNP markers in the present study while the majority of the markers in Hanley *et al*. (2006) were AFLPs.

Three of the five inversions we found support for involved poplar LG I and XVI and willow LG Ib. It thus appears that poplar LG I has been exposed to more changes, both inter- and intrachromosomal than any other linkage group. The analysis of the poplar genome [9] revealed that poplar LG I is a result of multiple rearrangements involving three tandem fusions, rearrangements that seem to have taken place before the evolution of modern poplar species as revealed by colinear genetic maps among multiple poplar species [9]. These rearrangements possibly took place during the genome-wide reorganization and diploidization following the whole-genome duplication (the Salicoid event) that took place before the divergence of *Salix *and *Populus *but after the split from *Arabidopsis *[9]. So, it seems that poplar LG I is the result of several changes occurring before the split of willow and poplar, and our results show that this linkage groups has been subjected to an elevated rate of rearrangements also after the divergence of *Salix *and *Populus*. One distinct feature of poplar LG I is that it contains significantly less euchromatin than any of the other 18 chromosomes, hence it is richer in heterochromatin and possibly repetitive DNA [9]. Since it is known that repetitive elements can trigger rearrangements this is one possible explanation to the many rearrangements involving LG I. It is also by far the largest LG in the willow map (279 cM). The same applies to its physical length, poplar I and XVI is together at least 42 Mb long (according to the poplar genome), which is much longer than any other poplar LG, in fact the second largest (LG II) is only ~23 Mb long (also according to the poplar genome sequence).

In addition to the above mentioned major rearrangements, seven markers had positions in willow and poplar that may reflect syntenic disparities between the genomes. They likely represent six different events of rearrangements because in two instances, markers are located next to each other and most likely reflect the same rearrangement events (II-23_sa and II-37_sa_pI are located next to each other). In summary, a surprisingly small numbers of markers show evidence for rearrangements between willow and poplar, most likely reflecting the stability of the willow and poplar genomes.

Using a divergence time of 45 my and counting the number of rearrangements between the poplar physical map and willow linkage maps (one fission, one fusion, five intrachromosomal inversions and six single markers that were located on different linkage groups in willow and poplar), we end up with a rate of 0.14 rearrangements/my/genome. Comparisons of these figures with estimates from other species should be done with caution as they depend strongly on the resolution of the map as well as the accuracy of the divergence estimates. With this in mind we can conclude that the rate of rearrangements in willow and poplar is at the lower end compared to estimates in other plant species, (0.17 - 0.6 rearrangements/My) [30,31]. The relatively few chromosomal rearrangements and greatly conserved gene order makes the use of poplar genomic resources for genetic and genomic works in willow very promising. This is of particular relevance for the identification of genes in willow QTLs with the help of the poplar genome.

### Sequence analysis of orthologus and paralogous loci in willow and poplar

Pairwise substitution rates in introns, K_i_, between orthologous and paralogous loci in willow and poplar were estimated in 115 gene fragments covering a total of 47966 nucleotides. K_i _between willow and poplar orthologs were approximately three times lower (median: 0.12) than between paralogs (willow - poplar paralogs: 0.42, poplar paralogs: 0.37). The two latter estimates did not differ significantly, suggesting that the two lineages have similar evolutionary rates since their divergence. The large difference in substitution rates between orthologs and paralogs was unexpected as the genome duplication and the divergence of the two genera have been suggested to have occurred within a short time span some 60 - 65 mya [9,32]. Our data clearly dispute this conclusion and instead suggest that the genome duplication occurred much earlier than the divergence of the two lineages.

According to interpretations of available fossil data, both *Salix *and *Populus *occur in middle Eocene sediments that are around 45 million years old [7,8]. Using this time as a proxy for the separation of the two lineages and K_i _= 0.12, the rate of substitution (r) was estimated to 1.28 × 10^-9 ^per site and year (T = K_i_/2r). Assuming that this substitution rate has been constant since the Salicoid duplication yields an estimated date of the genome duplication some 150 mya. This date is obviously much older than previous estimates, and is not consistent with other data showing that the Salicoid duplication is not shared with *Arabidopsis *[9]. Available data suggest that the lineages leading to *Arabidopsis *(Eurosids II) and Salicaceae (Eurosides I) separated 100 - 120 mya [33]. Assuming instead that the duplication occurred soon after the divergence of Eurosids I and Eurosids II at 110 mya yields an estimate of the divergence between *Salix *and *Populus *of around 40 mya. Considering the uncertainty in the fossil record of Salicaceae [8,34] such a date is perhaps not inconceivable. Dating of duplication and divergence events is notoriously difficult due to uncertainties both in the fossil data and substitution rates. Acknowledging these uncertainties, our data still suggests that the Salicoid genome duplication occurred considerably earlier than previously suggested. In a recent study, the poplar genome duplication was estimated to have occurred 45 mya, although it was acknowledged this was an underestimation of the timing due to the much slower substitution rates in poplar compared to other species [35].

Our data indicate substitution rates in Salicaceae between 1.28 × 10^-9 ^and 1.68 × 10^-9 ^per site and year depending on choice of calibration date, which is close to estimates for conifers (0.7 - 1.3 × 10^-9 ^[36]), but considerably lower than for annual plants with a reported range of 5 - 33 × 10^-9 ^[37-39]. These results are in line with the generally lower rates of substitutions estimated in perennials as opposed to annuals [40].

## Conclusions

In this study we have developed a large number of new SNP markers in willow by using the poplar genome sequence as a reference and genotyped them in two large mapping populations. Besides providing insights into the evolution of Salicaceae genomes, our maps will play a fundamental role in our quest to identify QTLs and candidate genes for traits important for biomass production, e.g. growth related traits, resistance to pathogens, drought tolerance and phenology (bud burst and cessation of growth). This is a prerequisite for the advancement of breeding through early marker-based selection. Although the genomes of willow and poplar appear well conserved, we have identified several previously unknown genome rearrangements. In addition, we have used a large data set of non-coding DNA sequences to estimate substitution rates in Salicaceae, which were similar to estimates found in other perennials but much slower than rates found in annuals. A conclusion is that the genomes evolve slowly both at the genomic levels and at the nucleotide level. We have also shown that the Salicoid genome duplication must have taken place much earlier than the separation of the two lineages, two events that were previously thought to have occurred within a short evolutionary time. Our data also highlights the difficulties in dating ancient whole genome duplication events, and that caution is necessary when using such estimates to draw conclusion about the evolutionary consequences of polyploidy.

## Methods

### Plant material and DNA extraction

The mapping population S_1 _with 463 F_1 _offspring was created by crossing a diploid hybrid male clone ('Björn'; *Salix viminalis × Salix schwerinii*) to a diploid *S. viminalis *female clone (78183). The *S. schwerinii *parent to Björn originates from Siberia while the *S. viminalis *parent as well as 78183 originates from southern Sweden. Initially, the parental clones to S_1 _were selected based on variation in phenology traits, but have also shown variation in rust resistance (*Melampsora *spp.) and different growth traits.

The mapping population S_3 _was made by crossing the diploid *S. viminalis *male clone (81084) with the diploid *S. viminalis *female clone (78195) to produce 282 F_1 _offspring. 78195 originates from Southwest of Sweden and 81084 originates from Southeast of Sweden. The parental clones to S_3 _were selected based on variation in gall midge resistance.

Genomic DNA was extracted from frozen young leaves with the FastDNA Kit (MP Biomedicals) according to the protocol provided with the kit and DNA concentrations were determined with a Nanodrop spectrophotometer (Nanodrop Technologies).

### SNP marker development and genotyping

SNP primers (Additional file 2) were designed to amplify fragments of genes at distances of approximately every 1 Mb on each linkage group (n = 19) of the poplar genome (The poplar genome website: http://genome.jgi-psf.org/Poptr1_1/Poptr1_1.home.html). In order to minimize amplification of multiple loci, genomic regions with little or no homology to other regions were selected. In effect, this means that single copy loci were favoured over those with multiple copies. Primers were positioned in exonic regions and designed to amplify across introns of approximately 500-1000 bp. Primers were also positioned to amplify parts of candidate genes for drought tolerance [41], rust [42-44] and insect resistance [45] (Additional file 1 and 2). 698 primer pairs were designed for the purpose of this study and PCR reactions were run with DNA from the parents of the mapping populations, of which 456 (65%) gave clean PCR products suitable for sequencing. 79 primer pairs were taken from Hanley *et al*. (2006) and tested in the parents as well, of which 70 resulted in clean PCR products. The PCR products were directly sequenced at Macrogen Inc. (Macrogen, Seoul, South Korea).

The primers were tested in each of the four parents in 15 μl volume PCR reactions containing 10 ng genomic DNA, 1 × PCR buffer II (Applied Biosystems), 2.5 mM MgCl_2 _(Applied Biosystems), 0.2 mM dNTP mix (Fermentas), 0.5 μM of each primer (Invitrogen), and 0.5 U AmpliTaq Gold DNA polymerase (Applied Biosystems). The PCR reactions were run on a MyCycler thermal cycler (Bio-Rad Laboratories) with a PCR profile consisting of 10 min denaturation at 95°C followed by 35 cycles of 30 s denaturation at 95°C, 30 s annealing at 55°C and 1 min extension at 72°C with a final 10 min 72°C step. Amplification success was determined by agarose gel electrophoresis. For the first 96 primer pairs, a poplar sample (*Populus trichocarpa*) was also included to test if failure to amplify a product in willow was due to differences in nucleotide sequence between willow and poplar and not a result of unsuccessful primer design.

The willow PCR products were cleaned with 1 μl of a mixture of Exonuclease I (New England BioLabs) and Shrimp Alkaline Phosphatase (SAP) (Fermentas) for every 5 μl of PCR product before they were sent for sequencing of both strands with the forward and reverse PCR primers. Sequences were aligned using the DNA Baser V. 2 software. Contigs were then examined for the presence of informative SNPs for mapping. The SNPs were genotyped in the S_1 _and S_3 _mapping populations using the Golden Gate Assay [46] from Illumina (San Diego) at the SNP Technology Platform, Uppsala University http://www.genotyping.se. We applied a nomenclature for the SNP markers with roman letters referring to the poplar linkage group the primers were designed to amplify, followed with an arbitrary number and the letters 'sa'. In case markers were informative only in one of the mapping populations or different SNPs were used in S_1 _and S_3 _populations, the suffix I or III was added to the name.

### Microsatellite and AFLP markers

PCR primers for microsatellite loci were taken from literature [47-51] and tested in the four parents (for details of the markers see Additional file 2). The PCR products were genotyped to check for levels of polymorphisms. The microsatellite loci selected for mapping were multiplexed and separated on ABI3730XL instruments at the Uppsala Genome Center. The majority of the microsatellite markers were PCR amplified by the Uppsala Genome Center, although some were amplified in-house. The in-house PCR reactions were run according to the protocol as described above for the SNP markers. The PCR reactions run at the Uppsala Genome Center were amplified in 10 μl volumes containing 10 ng genomic DNA, 1 × PCR buffer, 5% DMSO, 0.2 mM dNTP mix, 0.02 μM forward primer, 0.2 μM reverse primer, 0.2 μM M13-tail, 0.005 U Hot Star DNA polymerase and 1.25 μg BSA. The touchdown PCR profile consisted of 5 min denaturation at 95°C, six cycles of 30 s denaturation at 94°C, 30 s annealing at 58 - 53°C (-1°C per cycle) and 30 s extension at 72°C, seven cycles of 30 s denaturation at 94°C, 30 s annealing at 53 - 50°C (-0,5°C per cycle) and 30 s extension at 72°C, 30 cycles of denaturation at 94°C, 30 s annealing at 50°C and 30 s extension at 72°C and finally extension at 72°C for 7 min. Microsatellites were scored using the Peak Scanner software V 1.0 (Applied Biosystems). The genotypic data from an AFLP analysis was included from an earlier study on a subset of 89 individuals from the S_1 _cross [21].

### Map construction

Map construction was performed with the JoinMap 3.0 software [52]. Linkage maps were constructed using a LOD (logarithm of odds threshold) of 5.0 for map S_1 _and a LOD of 4.0 for map S_3 _to determine marker groupings. The LOD scores were selected based on the number of groups formed for each LOD score. The Kosambi mapping function was used for map construction with the following JoinMap settings: Rec = 0.4, LOD = 1.0, Jump = 5. The resulting linkage maps were drawn using the MapChart 2.1 software [53]. We did not use the ability of the software to force problematic markers on to the map and used the map resulting after the second round of map building. Mendelian segregations of the different markers were tested with chi-square analyses. We constructed S_1 _maps both with and without the AFLP markers but present the map including AFLPs.

The locations of the SNP markers developed in this study (markers with prefix 'sa') on the physical map of the poplar genome were placed according to the positions of the genes where the primers were designed. Since we did not have access to the target positions in the poplar genome of the markers taken from Hanley *et al*. (2006), the positions of these markers on the poplar physical map were determined by BLAST searching the poplar genome with the willow gene fragments.

### Database searches and Sequence analyses

BLAST searches against the *Populus trichocarpa *genome database on the NCBI web site were performed with the willow gene fragments that had been sequenced for the purpose of SNP identification in this study. BLAST searches were also performed with the poplar genomic sequences of the genes where the primers were positioned (paralog 1), hence the expected ortholog to the willow sequences. Usually we found two strong hits, of which we assumed the second best BLAST hit was the paralogous gene copy resulting from the *Populus *whole genome duplication (paralog 2). Since when designing the primers, genes or genomic regions with multiple copies were avoided as much as possible, a second BLAST hit was many times not present. When a second BLAST hit was present, the positions of the gene fragment of the willow sequence, the poplar paralog 1 and 2 in the poplar genome were compared, to determine if the expected orthologous gene fragment had been amplified in willow or, if the paralog had been amplified.

When three sequences were present for a gene-fragment; i.e. the willow sequence, the poplar paralog 1 and paralog 2, alignments were constructed using Clustal W [54] in the Alignment Explorer tool in MEGA4 using default parameters [55]. With the aim of comparing the number of substitutions in introns (K_i_) between orthologs and paralogs, pairwise K_i _was estimated in orthologous loci between willow and poplar, paralogous loci between willow and poplar and paralogous loci in poplar using MEGA4 with the maximum composite likelihood method. When the willow gene-fragment showed higher sequence homology to poplar paralog 2 and the location in the willow map correspond to the position of poplar paralog 2, willow and paralog 2 were regarded as orthologs. Frequency distributions of K_i _values for each pairwise comparison are presented in Figure 3. K_i _values were statistically compared between the three groups by non-parametric Mann-Whitney U tests in Mini-Tab 15. Medians and means (± standard error of the means) are presented.

## Authors' contributions

All authors have read and approved the final manuscript. SB designed the study, developed the markers, supervised the lab work, performed the analyses and drafted the manuscript. UL assisted in the analyses and drafting of the manuscript. SvA obtained funds and participated in the coordination of the study. TÖ performed the SNP genotyping. ACRW conceived to the study, obtained funds, participated in the design and coordination of the study and helped to analyze and draft the manuscript.

## Supplementary Material

Additional file 1 Markers located in candidate genes along with references Click here for file

Additional file 2 Genomic locations, primer sequences and segregation patterns for markers in the willow linkage maps Click here for file

Additional file 3 The consensus S_3 _linkage map aligned to the consensus S_1 _linkage map Click here for file

## References

[B1] Coghlan A Eichler EE Oliver SG Paterson AH Stein L Chromosome evolution in eukaryotes: a multi-kingdom perspective Trends Genet 2005 21 12 673 682 10.1016/j.tig.2005.09.009 16242204

[B2] Bennetzen JL Patterns in grass genome evolution Curr Opin Plant Biol 2007 10 2 176 181 10.1016/j.pbi.2007.01.010 17291821

[B3] Semon M Wolfe KH Rearrangement rate following the whole-genome duplication in teleosts Mol Biol Evol 2007 24 3 860 867 10.1093/molbev/msm003 17218642

[B4] Bhutkar A Schaeffer SW Russo SM Xu M Smith TF Gelbart WM Chromosomal rearrangement inferred from comparisons of 12 *Drosophila *genomes Genetics 2008 179 3 1657 1680 10.1534/genetics.107.086108 18622036 PMC2475759

[B5] Li WH Molecular evolution 1997 Sunderland, Mass: Sinauer Associates Inc

[B6] Studer RA Robinson-Rechavi M How confident can we be that orthologs are similar, but paralogs differ? Trends Genet 2009 25 5 210 216 10.1016/j.tig.2009.03.004 19368988

[B7] Boucher LD Manchester SR Judd WS An extinct genus of Salicaceae based on twigs with attached flowers fruits, and foliage from the Eocene Green River Formation of Utah and Colorado, USA Am J Bot 2003 90 9 1389 1399 10.3732/ajb.90.9.1389 21659238

[B8] Manchester SR Judd WS Handley B Foliage and fruits of early poplars (Salicaceae: *Populus*) from the eocene of Utah, Colorado, and Wyoming Int J Plant Sci 2006 167 4 897 908 10.1086/503918

[B9] Tuskan GA Difazio S Jansson S Bohlmann J Grigoriev I Hellsten U Putnam N Ralph S Rombauts S Salamov A Schein J Sterck L Aerts A Bhalerao RR Bhalerao RP Blaudez D Boerjan W Brun A Brunner A Busov V Campbell M Carlson J Chalot M Chapman J Chen GL Cooper D Coutinho PM Couturier J Covert S Cronk Q The genome of black cottonwood, *Populus trichocarpa *(Torr. & Gray) Science 2006 313 5793 1596 1604 10.1126/science.1128691 16973872

[B10] Bradshaw HD Villar M Watson BD Otto KG Stewart S Stettler RF Molecular genetics of growth and development in *Populus*. 3. A genetic linkage map of a hybrid poplar composed of RFLP, STS, and RAPD Markers Theor Appl Genet 1994 89 2-3 167 178 10.1007/BF00225137 24177824

[B11] Wu RL Han YF Hu JJ Fang JJ Li L Li ML Zeng ZB An integrated genetic map of *Populus deltoides *based on amplified fragment length polymorphisms Theor Appl Genet 2000 100 8 1249 1256 10.1007/s001220051431

[B12] Cervera MT Storme V Ivens B Gusmao J Liu BH Hostyn V Van Slycken J Van Montagu M Boerjan W Dense genetic linkage maps of three *Populus *species (*Populus deltoides*, *P. nigra *and *P. trichocarpa*) based on AFLP and microsatellite markers Genetics 2001 158 2 787 809 11404342 10.1093/genetics/158.2.787 PMC1461694

[B13] Yin T Zhang X Huang M Wang M Zhuge Q Tu S Zhu LH Wu R Molecular linkage maps of the *Populus *genome Genome 2002 45 3 541 555 10.1139/g02-013 12033623

[B14] Yin TM DiFazio SP Gunter LE Riemenschneider D Tuskan GA Large-scale heterospecific segregation distortion in *Populus *revealed by a dense genetic map Theor Appl Genet 2004 109 3 451 463 10.1007/s00122-004-1653-5 15168022

[B15] Woolbright SA Difazio SP Yin T Martinsen GD Zhang X Allan GJ Whitham TG Keim P A dense linkage map of hybrid cottonwood (*Populus fremontii *× *P. angustifolia*) contributes to long-term ecological research and comparison mapping in a model forest tree Heredity 2008 100 1 59 70 10.1038/sj.hdy.6801063 17895905

[B16] Hertzberg M Aspeborg H Schrader J Andersson A Erlandsson R Blomqvist K Bhalerao R Uhlen M Teeri TT Lundeberg J Sundberg B Nilsson P Sandberg G A transcriptional roadmap to wood formation Proc Natl Acad Sci USA 2001 98 25 14732 14737 10.1073/pnas.261293398 11724959 PMC64750

[B17] Azaiez A Boyle B Levee V Seguin A Transcriptome profiling in hybrid poplar following interactions with *Melampsora *rust fungi Mol Plant-Microbe Interact 2009 22 2 190 200 10.1094/MPMI-22-2-0190 19132871

[B18] Karp A Shield I Bioenergy from plants and the sustainable yield challenge New Phytol 2008 179 1 15 32 10.1111/j.1469-8137.2008.02432.x 18422906

[B19] Gillespie JH Population genetics: a concise guide 2004 2 Baltimore, Maryland, U.S.: The John Hopkins University Press

[B20] Hanley S Barker A Van Ooijen W Aldam C Harris L Åhman I Larsson S Karp A A genetic linkage map of willow (*Salix viminalis*) based on AFLP and microsatellite markers Theor Appl Genet 2002 105 6-7 1087 1096 10.1007/s00122-002-0979-0 12582937

[B21] Tsarouhas V Gullberg U Lagercrantz U An AFLP and RFLP linkage map and quantitative trait locus (QTL) analysis of growth traits in *Salix* Theor Appl Genet 2002 105 2-3 277 288 10.1007/s00122-002-0918-0 12582530

[B22] Rönnberg-Wästljung AC Tsarouhas V Semerikov V Lagercrantz U A genetic linkage map of a tetraploid *Salix viminalis *× *S. dasyclados *hybrid based on AFLP markers For Genet 2003 10 3 185 194

[B23] Hanley SJ Mallott MD Karp A Alignment of a *Salix *linkage map to the *Populus *genomic sequence reveals macrosynteny between willow and poplar genomes Tree Genet Genom 2006 3 1 35 48 10.1007/s11295-006-0049-x

[B24] Barcaccia G Meneghetti S Albertini E Triest L Lucchin M Linkage mapping in tetraploid willows: segregation of molecular markers and estimation of linkage phases support an allotetraploid structure for *Salix alba *× *Salix fragilis *interspecific hybrids Heredity 2003 90 2 169 180 10.1038/sj.hdy.6800213 12634824

[B25] Tsarouhas V Gullberg U Lagercrantz U Mapping of quantitative trait loci controlling timing of bud flush in *Salix* Hereditas 2003 138 3 172 178 10.1034/j.1601-5223.2003.01695.x 14641480

[B26] Tsarouhas V Gullberg U Lagercrantz U Mapping of quantitative trait loci (QTLs) affecting autumn freezing resistance and phenology in *Salix* Theor Appl Genet 2004 108 7 1335 1342 10.1007/s00122-003-1544-1 14747916

[B27] Rönnberg-Wästljung AC Glynn C Weih M QTL analyses of drought tolerance and growth for a *Salix dasyclados *× *Salix viminalis *hybrid in contrasting water regimes Theor Appl Genet 2005 110 3 537 549 10.1007/s00122-004-1866-7 15619077

[B28] Weih M Rönnberg-Wästljung AC Glynn C Genetic basis of phenotypic correlations among growth traits in hybrid willow (*Salix dasyclados *× *Salix viminalis*) grown under two water regimes New Phytol 2006 170 3 467 477 10.1111/j.1469-8137.2006.01685.x 16626469

[B29] Zhou F Xu Y RepPop: a database for repetitive elements in *Populus trichocarpa* BMC Genomics 2009 10 14 10.1186/1471-2164-10-14 19134208 PMC2645430

[B30] Lagercrantz U Comparative mapping between *Arabidopsis thaliana *and *Brassica nigra *indicates that *Brassica *genomes have evolved through extensive genome replication accompanied by chromosome fusions and frequent rearrangements Genetics 1998 150 3 1217 1228 9799273 10.1093/genetics/150.3.1217 PMC1460378

[B31] Kuittinen H de Haan AA Vogl C Oikarinen S Leppala J Koch M Mitchell-Olds T Langley CH Savolainen O Comparing the linkage maps of the close relatives *Arabidopsis lyrata *and *A. thaliana* Genetics 2004 168 3 1575 1584 10.1534/genetics.103.022343 15579708 PMC1448766

[B32] Jansson S Douglas CJ *Populus*: a model system for plant biology Annu Rev Plant Biol 2007 58 435 458 10.1146/annurev.arplant.58.032806.103956 17280524

[B33] Wikstrom N Savolainen V Chase MW Evolution of the angiosperms: calibrating the family tree Proc R Soc Biol Sci Ser B 2001 268 1482 2211 2220 10.1098/rspb.2001.1782 PMC1088868 11674868

[B34] Forest F Calibrating the Tree of Life: fossils, molecules and evolutionary timescales Ann Bot 2009 104 5 789 794 10.1093/aob/mcp192 19666901 PMC2749537

[B35] Fawcett JA Maere S Peer Y Van de Plants with double genomes might have had a better chance to survive the Cretaceous-Tertiary extinction event Proc Natl Acad Sci USA 2009 106 14 5737 5742 10.1073/pnas.0900906106 19325131 PMC2667025

[B36] Willyard A Syring J Gernandt DS Liston A Cronn R Fossil calibration of molecular divergence infers a moderate mutation rate and recent radiations for *Pinus* Mol Biol Evol 2007 24 1 90 101 10.1093/molbev/msl131 16997907

[B37] Koch MA Haubold B Mitchell-Olds T Comparative evolutionary analysis of chalcone synthase and alcohol dehydrogenase loci in *Arabidopsis*, *Arabis*, and related genera (Brassicaceae) Mol Biol Evol 2000 17 10 1483 1498 11018155 10.1093/oxfordjournals.molbev.a026248

[B38] Gaut BS Morton BR McCaig BC Clegg MT Substitution rate comparisons between grasses and palms: Synonymous rate differences at the nuclear gene *Adh *parallel rate differences at the plastid gene *rbcL* Proc Natl Acad Sci USA 1996 93 19 10274 10279 10.1073/pnas.93.19.10274 8816790 PMC38374

[B39] Clark RM Tavare S Doebley J Estimating a nucleotide substitution rate for maize from polymorphism at a major domestication locus Mol Biol Evol 2005 22 11 2304 2312 10.1093/molbev/msi228 16079248

[B40] Smith SA Donoghue MJ Rates of molecular evolution are linked to life history in flowering plants Science 2008 322 5898 86 89 10.1126/science.1163197 18832643

[B41] Street NR Skogstrom O Sjodin A Tucker J Rodriguez-Acosta M Nilsson P Jansson S Taylor G The genetics and genomics of the drought response in *Populus* Plant J 2006 48 3 321 341 10.1111/j.1365-313X.2006.02864.x 17005011

[B42] Kohler A Rinaldi C Duplessis S Baucher M Geelen D Duchaussoy F Meyers BC Boerjan W Martin F Genome-wide identification of *NBS *resistance genes in *Populus trichocarpa* Plant Mol Biol 2008 66 6 619 636 10.1007/s11103-008-9293-9 18247136

[B43] Yin TM DiFazio SP Gunter LE Jawdy SS Boerjan W Tuskan GA Genetic and physical mapping of *Melampsora *rust resistance genes in *Populus *and characterization of linkage disequilibrium and flanking genomic sequence New Phytol 2004 164 1 95 105 10.1111/j.1469-8137.2004.01161.x 33873470

[B44] Rinaldi C Kohler A Frey P Duchaussoy F Ningre N Couloux A Wincker P Le Thiec D Fluch S Martin F Duplessis S Transcript profiling of poplar leaves upon infection with compatible and incompatible strains of the foliar rust *Melampsora larici-populina* Plant Physiol 2007 144 1 347 366 10.1104/pp.106.094987 17400708 PMC1913798

[B45] Ralph SG Chun HJ Cooper D Kirkpatrick R Kolosova N Gunter L Tuskan GA Douglas CJ Holt RA Jones SJ Marra MA Bohlmann J Analysis of 4,664 high-quality sequence-finished poplar full-length cDNA clones and their utility for the discovery of genes responding to insect feeding BMC Genomics 2008 9 57 10.1186/1471-2164-9-57 18230180 PMC2270264

[B46] Fan JB Oliphant A Shen R Kermani BG Garcia F Gunderson KL Hansen M Steemers F Butler SL Deloukas P Galver L Hunt S McBride C Bibikova M Rubano T Chen J Wickham E Doucet D Chang W Campbell D Zhang B Kruglyak S Bentley D Haas J Rigault P Zhou L Stuelpnagel J Chee MS Highly parallel SNP genotyping Cold Spring Harbor Symp Quant Biol 2003 68 69 78 10.1101/sqb.2003.68.69 15338605

[B47] Rahman MH Dayanandan S Rajora OP Microsatellite DNA markers in *Populus tremuloides* Genome 2000 43 2 293 297 10.1139/gen-43-2-293 10791817

[B48] Schoot J van der Pospiskova M Vosman B Smulders MJM Development and characterization of microsatellite markers in black poplar (*Populus nigra *L.) Theor Appl Genet 2000 101 1-2 317 322 10.1007/s001220051485

[B49] Lian C Nara K Nakaya H Zhou Z Wu B Miyashita N Hogetsu T Development of microsatellite markers in polyploid *Salix reinii* Mol Ecol Notes 2001 1 3 160 161 10.1046/j.1471-8278.2001.00059.x

[B50] Barker JHA Pahlich A Trybush S Edwards KJ Karp A Microsatellite markers for diverse *Salix *species Mol Ecol Notes 2003 3 1 4 6 10.1046/j.1471-8286.2003.00332.x

[B51] Hanley SJ Genetic mapping of important agronomic traits in biomass willow PhD thesis 2003 University of Bristol, U.K., Department of Agricultural Science

[B52] Van Ooijen JW Voorrips RE JoinMap 3.0, Software for the calculation of genetic linkage maps 2001 Plant Research International, Wageningen, The Netherlands

[B53] Voorrips RE MapChart: software for the graphical presentation of linkage maps and QTLs J Hered 2002 93 1 77 78 10.1093/jhered/93.1.77 12011185

[B54] Thompson JD Higgins DG Gibson TJ CLUSTAL W: improving the sensitivity of progressive multiple sequence alignment through sequence weighting, position-specific gap penalties and weight matrix choice Nucleic Acids Res 1994 22 22 4673 4680 10.1093/nar/22.22.4673 7984417 PMC308517

[B55] Tamura K Dudley J Nei M Kumar S MEGA4: Molecular Evolutionary Genetics Analysis (MEGA) software version 4.0 Mol Biol Evol 2007 24 8 1596 1599 10.1093/molbev/msm092 17488738

