# Supplementary material for: High-density linkage mapping and evolution of paralogs and orthologs in Salix and Populus
Source: BMC Genomics. 2010 Feb 23;11:129. doi: 10.1186/1471-2164-11-129 (PMC2834636; doi:10.1186/1471-2164-11-129)
Supplement: Additional file 1 — Markers located in candidate genes along with references [file 1471-2164-11-129-S1.PDF]

Additional file 1. Markers located in candidate genes along with references.

Markers	Reference	PU number *	Protein ID **	Genbank ID ***
I_DT_11_sa	Street *et al.* 2006	PU07050		CK092601
I_DT_25_sa_pI+III	Street *et al.* 2006	PU11545		CK095408
I_DT_27_sa	Street *et al.* 2006	PU09275		CK089980
V_DT_5_sa	Street *et al.* 2006	PU04104		BU820207
VI_DT_12_sa	Street *et al.* 2006	PU12091		CK101003
VI_DT_20_sa_pI+III	Street *et al.* 2006	PU07550		CK103196
X_DT_23_sa	Street *et al.* 2006	PU10422		BI123582
X_DT_28_sa	Street *et al.* 2006	PU10560		BI123331
XVI_DT_4_sa	Street *et al.* 2006	PU10578		CK094405
DT_6_sa	Street *et al.* 2006	PU12988		CK091511
Ro_1_sa	Kohler *et al.* 2008		582100	
Ro_3_sa_pI+III	Kohler *et al.*		249428	

	2008		
Ro_9_sa	Kohler *et al.* 2008	594667	
Ro_4b_sa	Kohler *et al.* 2008	764595	
Ro_11_sa	Yin *et al.* 2004	800364	
Ro_16_sa_pI	Rinaldi *et al.* 2007	417599	
Ro_18_sa_pI	Rinaldi *et al.* 2007	669475	
Ro_27_sa	Rinaldi *et al.* 2007	828962	
Ro_28_sa_pI+III	Rinaldi *et al.* 2007	659435	
R_4_sa	Ralph *et al.* 2008		EF146918
R_9_sa	Ralph *et al.* 2008		EF144899
R_23_sa	Ralph *et al.* 2008		EF147658
R_24_sa	Ralph *et al.* 2008		EF144759
R_28_sa	Ralph *et al.* 2008		EF147174

R_29_sa	Ralph *et al.* 2008	EF148579
R_32_sa_pI	Ralph *et al.* 2008	EF147184
R_33_sa_pI+III	Ralph *et al.* 2008	EF147117
R_36_sa	Ralph *et al.* 2008	EF148121
R_41_sa_pI	Ralph *et al.* 2008	EF148393
R_48_sa	Ralph *et al.* 2008	EF147179
R_51_sa	Ralph *et al.* 2008	EF144570
R_52_sa	Ralph *et al.* 2008	EF148589
R_54_sa	Ralph *et al.* 2008	EF145764
R_56_sa	Ralph *et al.* 2008	EF144919
R_59_sa	Ralph *et al.* 2008	EF145551
R_60_sa_pI	Ralph *et al.* 2008	EF145493
R_61_sa	Ralph *et al.*	EF145102

	2008		
R_62_sa	Ralph *et al.* 2008		EF147330
R_66_sa	Ralph *et al.* 2008		EF146705
R_67_sa_pI+III	Ralph *et al.* 2008		EF146405
R_69_sa	Ralph *et al.* 2008		EF144380
R_71_sa	Ralph *et al.* 2008		EF148294
R_73_sa	Ralph *et al.* 2008		EF145159
R_74_sa	Ralph *et al.* 2008		EF144553
R_76_sa	Ralph *et al.* 2008		EF148737
R_79_sa	Ralph *et al.* 2008		EF147535
R_80_sa	Ralph *et al.* 2008		EF146002
R_84_sa	Ralph *et al.* 2008		EF148221
Ph2		566667	
Ph5		826816	

Ph14_III	833761
Ph17	773175
Ph18	1083038
Ph21	876150
Ph27	560536
Ph30	556324
Ph32	706232

* PU number found at PopulusDB http://www.populus.db.umu.se/

** Protein ID found at http://genome.jgi-psf.org/Poptr1_1/Poptr1_1.home.html

*** Genbank ID found at http://www.ncbi.nlm.nih.gov/

References

Kohler A, Rinaldi C, Duplessis S, Baucher M, Geelen D, Duchaussoy F, Meyers BC, Boerjan W, Martin F: Genome-wide identification of NBS resistance genes in *Populus trichocarpa*. Plant Mol Biol 2008, 66(6):619-636.

Ralph SG, Chun HJ, Cooper D, Kirkpatrick R, Kolosova N, Gunter L, Tuskan GA, Douglas CJ, Holt RA, Jones SJ et al: Analysis of 4,664 high-quality sequence-finished poplar full-length cDNA clones and their utility for the discovery of genes responding to insect feeding. BMC Genomics 2008, 9:57.

Rinaldi C, Kohler A, Frey P, Duchaussoy F, Ningre N, Couloux A, Wincker P, Le Thiec D, Fluch S, Martin F et al: Transcript profiling of poplar leaves upon infection with compatible and incompatible strains of the foliar rust *Melampsora larici-populina*. Plant Physiol 2007, 144(1):347-366.

Street NR, Skogstrom O, Sjodin A, Tucker J, Rodriguez-Acosta M, Nilsson P, Jansson S, Taylor G: The genetics and genomics of the drought response in *Populus*. Plant J 2006, 48(3):321-341.

Yin TM, DiFazio SP, Gunter LE, Jawdy SS, Boerjan W, Tuskan GA: Genetic and physical mapping of Melampsora rust resistance genes in *Populus* and characterization of linkage disequilibrium and flanking genomic sequence. New Phytol 2004, 164(1):95-105.
