# Supplementary figures and images for: High-density linkage mapping and evolution of paralogs and orthologs in Salix and Populus
Source: BMC Genomics. 2010 Feb 23;11:129. doi: 10.1186/1471-2164-11-129 (PMC2834636; doi:10.1186/1471-2164-11-129)

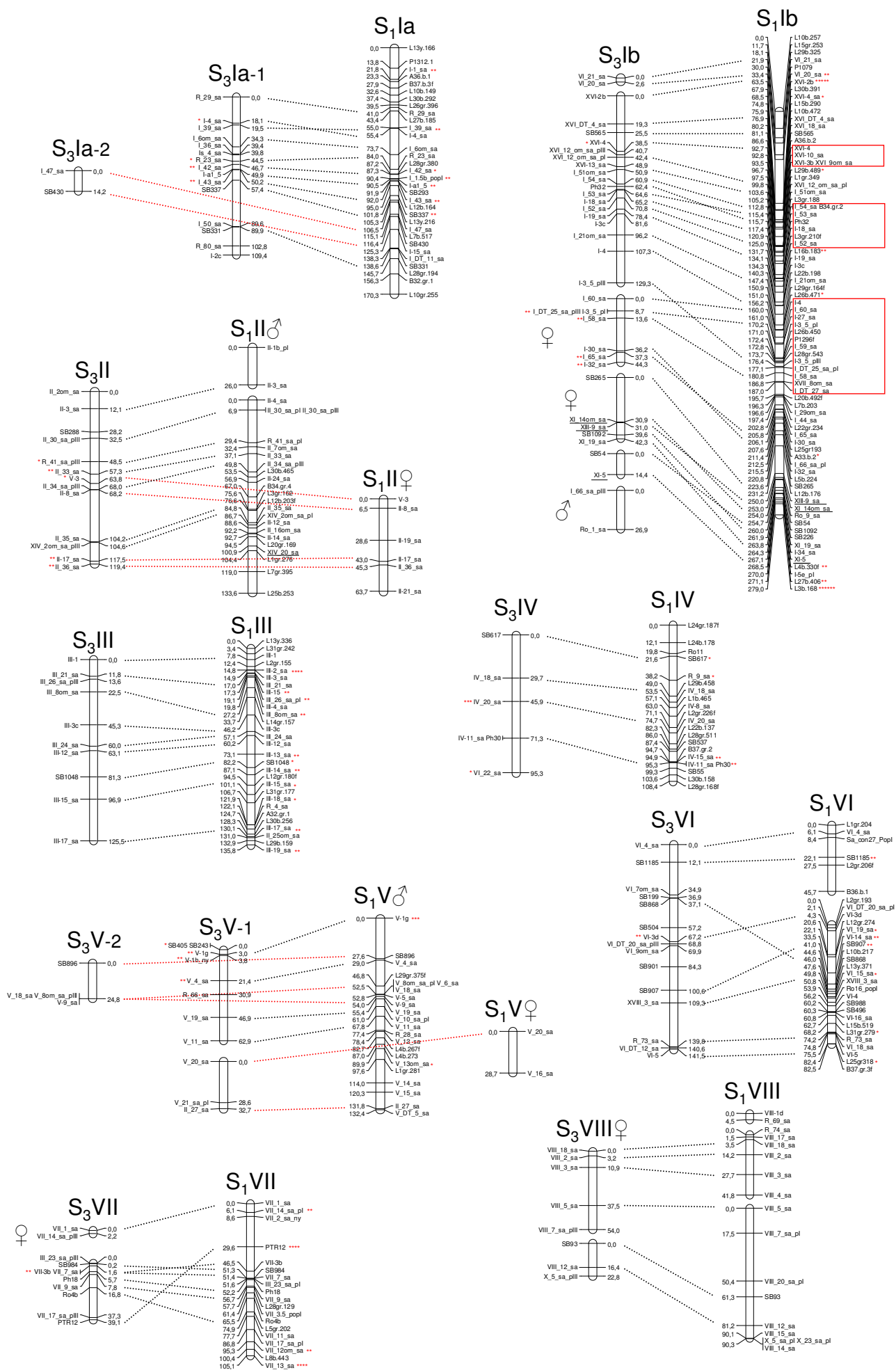

Supplement: Additional file 3 — The consensus S3 linkage map aligned to the consensus S1 linkage map [file 1471-2164-11-129-S3.PDF]
